# The potential of deferasirox as a novel therapeutic modality in gastric cancer

**DOI:** 10.1186/s12957-016-0829-1

**Published:** 2016-03-10

**Authors:** Jung Hye Choi, Jung Soon Kim, Young Woong Won, Jieun Uhm, Byeong Bae Park, Young Yiul Lee

**Affiliations:** Department of Internal Medicine, Hanyang University Guri Hospital, Hanyang University College of Medicine, Guri-si, Gyeonggi-do 471-701 Republic of Korea

**Keywords:** Deferasirox, Stomach neoplasm, Cisplatin

## Abstract

**Background:**

Iron is a crucial element for cell proliferation, growth, and metabolism. However, excess iron and altered iron metabolism are both associated with tumor initiation and tumor growth. Deferasirox is an oral iron chelator. Although some studies have indicated that deferasirox is a promising candidate for anti-cancer therapies, its effectiveness against gastric cancer has not yet been determined. This study was conducted to determine whether deferasirox exerts anti-tumor effects in gastric cancer cell lines and whether deferasirox and cisplatin act synergistically.

**Methods:**

Four human gastric cancer cell lines (AGS, MKN-28, SNU-484, and SNU-638) were treated with various concentrations of deferasirox to determine the IC_50_ for each cell line. The effects of deferasirox on the cell cycle were evaluated by flow cytometry, and the effects of deferasirox on iron metabolism, the cell cycle, and apoptosis were assessed by Western blotting. To determine whether deferasirox enhances the effect of cisplatin, AGS cells were cultured in the presence and absence of cisplatin.

**Results:**

Deferasirox inhibited the proliferation of all gastric cancer cell lines as assessed by MTT assays. Since the IC_50_ of deferasirox was the lowest (below 10 μM) in AGS cells, subsequent experiments were performed in this line. Deferasirox upregulated transferrin receptor 1 expression and decreased ferroportin expression. Moreover, deferasirox induced G1 arrest; upregulated p21, p27, and p53 expression; and downregulated cyclin D1, cyclin B, and CDK4 expression. Furthermore, deferasirox induced apoptosis, upregulated *N-myc* downstream regulated gene 1 (NDRG1), and downregulated p-mTOR and c-myc expression. It was also found to act synergistically with cisplatin.

**Conclusions:**

Our results suggest that deferasirox may exert anti-tumor effects in the context of gastric cancer. Deferasirox affects a number of different pathways and molecules; for instance, deferasirox upregulates NDRG1 expression, inhibits the cell cycle, downregulates mTOR and c-myc expression, and induces apoptosis. In addition, deferasirox appears to potentiate the anti-cancer effects of cisplatin. Although the efficacy of deferasirox remains to be tested in future studies, the results presented here indicate that deferasirox is a promising novel anti-cancer therapeutic agent.

## Background

Gastric cancer is one of the leading causes of cancer-related deaths in Korea [1]. Although patients with gastric cancer show excellent outcomes if the cancer is detected early, inoperable advanced and recurrent gastric cancers are still associated with poor survival rates. In the past few decades, substantial improvements in chemotherapeutic agents have improved survival in advanced gastric cancer. Recently, overall survival was significantly prolonged in patients with HER2-positive advanced gastric or gastro-esophageal junction cancer by treating with trastuzumab (HER-2 monoclonal antibody) in combination with conventional chemotherapy [2]. However, the overall survival was only 13.8 months. Therefore, new agents are urgently required.

Iron is an essential element for cell proliferation, growth, and metabolism. However, excess iron and altered iron metabolism have been associated with tumor initiation and tumor growth [3]. Epidemiological studies have revealed that a high iron intake is associated with an increased risk of colorectal cancer [4]. Many cancer cells alter iron metabolism because malignant cells require more iron than normal cells. To increase the labile iron pool, cancer cells have been shown to upregulate the expression of transferrin receptor 1 (TFR1) and hepcidin, in addition to downregulating ferroportin expression [3].

Deferasirox (Exjade®), an oral tridentate iron (Fe^3+^) chelator, is rapidly absorbed from the gut and has a relatively long half-life (8 to 16 h). Thus, once-daily dosing can achieve sustained circulating drug levels sufficient for the scavenging of non-transferrin-bound plasma iron. Although deferasirox has been associated with some adverse effects such as gastrointestinal disturbance, skin rash, and renal toxicity, it is relatively well tolerated. Therefore, deferasirox is currently the most commonly used iron chelator for the treatment of iron overload disease [5].

Recently, several studies have investigated the potential of deferasirox as an anti-neoplastic agent. Deferasirox has been reported to inhibit NF-κB activity in blood samples from patients with myelodysplastic syndrome and in leukemia cell lines [6]; moreover, deferasirox was also shown to repress the mTOR pathway in myeloid leukemia cells [7]. Regarding clinical data, one case report showed that deferasirox treatment achieved complete remission in patients with chemotherapy-refractory acute monocytic leukemia [8]. Furthermore, post hoc analysis of a multicenter trial revealed that deferasirox improved hematological parameters in patients with myelodysplastic syndrome [9]. At present, most reports of deferasirox as an anti-neoplastic agent have been in hematologic malignancies; only a few studies have focused on solid tumors. Recently, deferasirox was shown to inhibit the growth of lung and esophageal cancer cells both in vitro and in vivo [10, 11]. However, the effect of deferasirox on gastric cancer has not yet been determined, and the mechanism by which deferasirox exerts its anti-tumor effects remains poorly understood. Therefore, this study was conducted to investigate whether deferasirox exerts anti-tumor effects on gastric cancer cell lines and also whether deferasirox acts synergistically with cisplatin.

## Methods

### Cell culture

Four human gastric cancer cell lines (AGS, MKN-28, SNU-484, and SNU-638) were obtained from the Korean Cell Line Bank. All cells were cultured in RPMI 1640 medium containing 10 % fetal bovine serum and antibiotics (100 U/mL penicillin and 100 μg/mL streptomycin) in a humidified 5 % CO_2_ incubator at 37 °C.

### Reagents and antibodies

Deferasirox (Exjade®) was donated by Novartis (Basel, Switzerland). Goat polyclonal anti-NDRG1 (*N-myc* downstream regulated gene 1) (catalog no. ab37897) and rabbit polyclonal anti-ferroportin (catalog no. ab85370) antibodies were purchased from Abcam (Cambridge, UK). Anti-TFR1 mouse monoclonal antibodies (catalog no. 136800) were obtained from Life Technologies (Carlsbad, CA, USA), and FeSO_4_ was purchased from Sigma-Aldrich (St. Louis, MO, USA). Anti-p53, anti-p27, p21, cyclin A, cyclin B, cyclin D1, cyclin E, CDK2, CDK4, CDK6, c-myc, pro-caspase 3, and BAX antibodies were purchased from Santa Cruz Biotechnology (Santa Cruz, CA, USA). Anti-p-mTOR and pro-caspase 8 antibodies were obtained from Cell Signaling Technology (Beverly, MA, USA).

### Growth inhibition assay

Growth inhibition was measured with MTT (3-[4,5-dimethylthiazol-2-yl]-2,5-diphenyltetrazolium bromide) as previously described [12]. Briefly, cells were seeded (2 × 10^3^ cells/well) in 96-well microtiter plates (Nunc, Roskilde, Denmark) and incubated at 37 °C for 24, 48, or 72 h. MTT solution (50 μL) from Sigma (2 mg/mL in PBS) was added to each well, and the plates were incubated for an additional 4 h at 37 °C. After this incubation, the MTT solution was aspirated off. To solubilize the formazan crystals formed in viable cells, 200 μL of DMSO was added to each well. The plates were shaken for 30 min at room temperature, and the absorbance of each well at 595 nm was read immediately with a scanning multiwell spectrophotometer (Bio-Rad, iMarkTM microplate reader).

To determine the concentration of deferasirox required to kill 50 % of the cells (IC_50_), AGS, MKN-28, SNU-484, and SNU-638 cells were treated with 0, 1, 10, 50, and 100 μM of deferasirox for 24, 48, and 72 h. These results were used to select the gastric cell line with the greatest sensitivity to deferasirox for all subsequent experiments.

### Cell cycle analysis

After 24-h incubation of AGS cells with 0, 10, and 100 μM of deferasirox at 37 °C, the cells were washed twice with PBS, fixed overnight with 70 % ethanol, washed with PBS, and stained with 50 μg/mL of propidium iodide (PI) containing RNase A at 50 μg/mL. The DNA contents of the cells (10,000 cells/experimental group) were analyzed using a FACSCanto II flow cytometer (Becton Dickinson, San Jose, CA, USA) equipped with BD FACSDivaTM software (v6.1.3). The percentages of the cell populations in each cell cycle phase (G1, S, or G2/M) were calculated from the DNA content histograms.

### Western blot analysis

AGS cells were incubated with 0, 10, and 100 μM of deferasirox at 37 °C for 24 h. The cells were washed with PBS, resuspended in lysis buffer [50 mM Tris (pH 7.5), 1 % NP-40, 2 mM EDTA, 10 mM NaCl, 20 μg/mL aprotinin, 20 μg/mL leupeptin, and 1 mM phenylmethylsulfonyl fluoride], and placed on ice for 20 min. Proteins in the lysates (20–30 μg) were resolved on 10–15 % SDS-polyacrylamide denaturing gels and transferred to nitrocellulose membranes for 90–120 min. Nonspecific binding sites were blocked with 5 % skim milk for 1 h, and the membranes were then incubated overnight with primary antibodies (all at a 1:1000 dilution). The antibodies and the related processes that were used to investigate were as follows: anti-TFR1 and anti-ferroportin for iron metabolism; anti-p53, p27, p21, cyclin A, cyclin B, cyclin D1, cyclin E, CDK2, CDK4, and CDK6 for the cell cycle; anti-pro-caspase 3, pro-caspase 8, pro-caspase 9, and BAX for apoptosis; anti-NDRG1 for metastasis; and anti-p-mTOR and c-myc. Immunoreactive bands were visualized with an ECL kit (Intron, Korea).

### Statistical analysis

Data are presented as means ± SEMs (error bars). Differences were analyzed with Student’s *t* test. *P* values <0.05 were considered statistically significant.

## Results

### Effect of deferasirox on the growth of gastric cancer cell lines

**Fig. 1:**
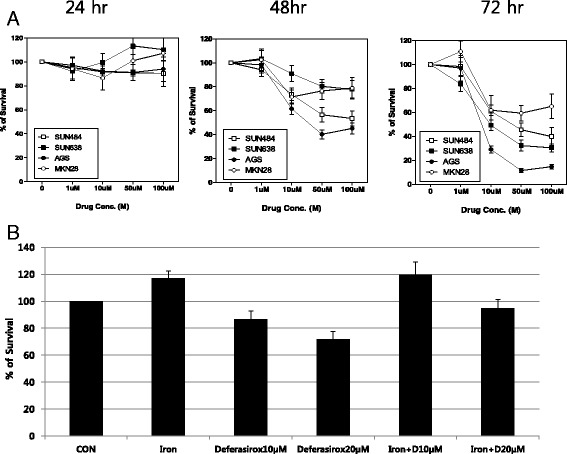
Inhibitory effect of deferasirox on the growth of gastric cancer cell lines. **a** Cell viability was measured by the MTT assay. AGS, MKN-28, SNU-484, and SNU-638 cells were incubated with 0, 1, 10, 50, and 100 μM of deferasirox at 37 °C for 24, 48, or 72 h. Deferasirox treatment resulted in dose-dependent and time-dependent growth inhibition in all four gastric cancer cell lines. **b** AGS cells were cultured with 10 and 20 μM of deferasirox either alone or in the presence of FeSO_4_ (100 μM) for 48 h. The inhibitory effect of deferasirox was reversed by FeSO_4_ supplement

The ability of deferasirox to inhibit the growth of the four gastric cancer cell lines was determined by an MTT proliferation assay. AGS, MKN-28, SNU-484, and SNU-638 cells were incubated with 0, 1, 10, 50, and 100 μM deferasirox at 37 °C for 24, 48, or 72 h. Deferasirox inhibited the growth of all four gastric cancer cell lines in a dose-dependent and time-dependent manner (Fig. 1a). Since the IC_50_ of deferasirox at 72 h was the lowest in AGS cells (less than 10 μM), all subsequent experiments were performed using these cells.

AGS cells were cultured with 10 and 20 μM of deferasirox either alone or in the presence of FeSO_4_ (100 μM) for 48 h. The inhibitory effect of deferasirox was reversed by FeSO_4_ supplement (Fig. 1b).

### Cell cycle analysis in AGS cells

**Fig. 2:**
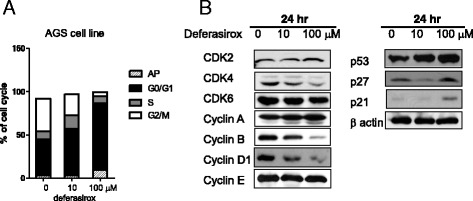
Effect of deferasirox on cell cycle progression in AGS cells. **a** AGS cells were incubated with 0, 10, and 100 μM of deferasirox at 37 °C for 24 h. Cell cycle progression was analyzed by FACS. Deferasirox treatment for 24 h led to a dose-dependent accumulation of AGS cells in G1 phase (41.8 % at 0 μM, 53.7 % at 10 μM, and 77.2 % at 100 μM). **b** Western blot analysis of cell cycle-related molecules showed that deferasirox upregulated p21, p27, and p53 and downregulated cyclin D1, cyclin B, and CDK4

Iron depletion induces G1/S arrest by affecting the expression of critical molecules for cell cycle progression such as cyclin D1 and p21 [13]. The effects of deferasirox on the cell cycle were determined by fluorescence-activated cell sorting (FACS) using propidium iodide. AGS cells were incubated with 0, 10, and 100 μM of deferasirox at 37 °C for 24 h. As shown in Fig. 2a, treatment of AGS cells with deferasirox for 24 h led to an accumulation of cells in G1 phase in a dose-dependent manner (41.8 % at 0 μM, 53.7 % at 10 μM, and 77.2 % at 100 μM). This result indicates that deferasirox induces G1 arrest. Western blot analysis of cell cycle-related proteins showed that deferasirox induced the upregulation of p21, p27, and p53, and the downregulation of cyclin D1, cyclin B, and CDK4 (Fig. 2b). These results suggest that the anti-proliferative effect of deferasirox is due to cell cycle inhibition.

### Effect of deferasirox on iron metabolism and other pathways

**Fig. 3:**
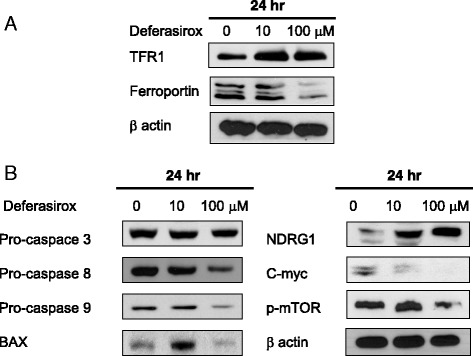
Effect of deferasirox on iron metabolism and other pathways. **a** Treatment with deferasirox for 24 h resulted in an increased level of TFR1 and a decreased level of ferroportin. **b** Deferasirox also induced apoptosis, upregulated NDRG1, and downregulated p-mTOR and c-myc as assessed by Western blot analysis

For iron uptake into the cell, circulating iron-transferrin complexes bind to the cell surface receptor TFR1. Iron exits via ferroportin, an iron efflux pump that is regulated by hepcidin. In cancer cells, TFR1 and hepcidin have been shown to be upregulated and ferroportin is downregulated, which cumulatively lead to increased concentrations of intracellular iron [3]. The effect of deferasirox on iron metabolism was evaluated by Western blot analysis of TFR1 and ferroportin. The level of TFR1 increased after 24 h of treatment with deferasirox. In contrast, ferroportin expression decreased (Fig. 3a). These results are consistent with those of previous studies [10, 11].

The effects of deferasirox on apoptosis were next evaluated by FACS and Western blot analysis of apoptosis-related proteins. As shown in Fig. 2a, AGS cells treated with deferasirox for 24 h exhibited an accumulation of cells in sub-G1 (apoptotic) phase (3.2 % at 0 μM, 3.3 % at 10 μM, and 9.5 % at 100 μM). Moreover, deferasirox treatment decreased the expression of pro-caspase 3, pro-caspase 8, and pro-caspase 9 and increased the expression of BAX (Fig. 3b). NDRG1 is known to be a suppressor of cell growth and metastasis. Deferasirox increased the level of NDRG1. In addition, c-myc and phospho-mTOR expression were decreased after 24 h of treatment with deferasirox (Fig. 3b). These results suggest that deferasirox induces apoptosis, inhibits distant metastasis, and suppresses the c-myc and mTOR pathways.

### Synergistic effect of deferasirox and cisplatin

**Fig. 4:**
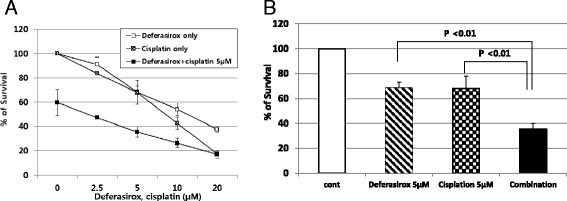
Synergistic effect of deferasirox and cisplatin. **a**, **b** AGS cells were treated with 0, 2.5, 5, 10, and 20 μM of deferasirox, either alone or in the presence of a fixed concentration of cisplatin (5 μM) for 48 h. AGS cells treated with deferasirox and cisplatin showed a significant decrease in cellular viability compared with cells treated with either deferasirox or cisplatin alone

To assess whether deferasirox could enhance the effect of cisplatin, AGS cells were cultured with or without cisplatin and their viability was determined using the MTT assay. Treatment with cisplatin for 48 h reduced the number of viable cells, with an IC_50_ of 5–10 μM. To determine whether deferasirox exerts a synergistic effect with cisplatin, AGS cells were treated with 0, 2.5, 5, 10, and 20 μM of deferasirox either alone or in the presence of a fixed concentration of cisplatin (5 μM) for 48 h. As shown in Fig. 4a, b, AGS cells treated with deferasirox and cisplatin showed a significantly greater decrease in cellular viability compared with cells treated with either deferasirox or cisplatin alone (*P* <0.01). These results suggest that deferasirox enhances cisplatin-mediated inhibition of AGS cell growth.

**Fig. 5:**
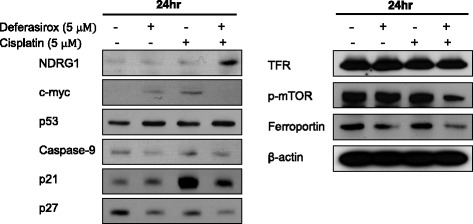
Molecular mechanisms of synergistic effect. Western blot analysis was performed with lysates of AGS cells treated with deferasirox (5 μM), cisplatin (5 μM), or both for 24 h. The combination of deferasirox and cisplatin induced the upregulation of NDRG1, p21, and p53, in addition to the downregulation of phospho-mTOR, ferroportin, and pro-caspase 9

To investigate the molecular mechanisms underlying this effect, Western blot analysis was used to assess the levels of various molecules in AGS cells treated with deferasirox (5 μM), cisplatin (5 μM), or both. The combination of deferasirox and cisplatin resulted in the upregulation of NDRG1, p21, and p53. In contrast, this combination resulted in the downregulation of phospho-mTOR, ferroportin, and pro-caspase 9. These findings suggest that deferasirox potentiates the anti-cancer effects of cisplatin through various pathways (Fig. 5).

## Discussion

In this study, we found that deferasirox inhibits the proliferation of gastric cancer cells. Deferasirox was also found to induce G1 arrest; upregulate p21, p27, and p53 expression; and downregulate cyclin D1, cyclin B, and CDK4 expression. Deferasirox also induced apoptosis, upregulated NDRG1, and downregulated p-mTOR and c-myc. These results suggest that deferasirox exerts anti-tumor effects in gastric cancer cells via various pathways. Specifically, our data indicate that deferasirox alters iron metabolism, inhibits cell cycle progression, affects mTOR signaling and metastasis pathways, and induces apoptosis. In addition, deferasirox appears to potentiate the anti-proliferative effect of cisplatin in stomach cancer cells.

Iron is essential for cell survival but can also cause cellular damage by generating reactive oxygen species [14]. Although the level of intracellular iron is tightly regulated in normal cells, the level of intracellular iron is elevated in cancer cells due to increased expression of TFR1 and hepcidin and reduced expression of ferroportin [3]. Since excess iron and altered iron metabolism can lead to tumor initiation and growth, iron chelators are believed to be promising anti-cancer agents. Several lines of evidence support the idea that iron chelators are potential anti-tumor therapeutics. Firstly, increased levels of intracellular iron are known to promote DNA synthesis. Since iron is essential for the activity of ribonucleotide reductase, a key enzyme for DNA synthesis, iron plays an important role in cell proliferation [15]. Therefore, increased iron is required to augment ribonucleotide reductase activity in neoplastic cells. Secondly, iron depletion can cause G1/S arrest and induce apoptosis [13]. Cyclin D1 binds to CDK4 and CDK6, thereby resulting in G1/S progression via phosphorylation of retinoblastoma protein (RB). This phosphorylation in turn results in the release of the transcription factor E2F from RB. Iron depletion is known to decrease cyclin D1 and CDK expression. Thirdly, excessive cellular iron can drive the Wnt signaling pathway, which is known to be important for tumor progression [16].

We performed this study to investigate whether deferasirox exerts anti-tumor effects in the context of gastric cancer. We chose deferasirox out of the numerous commercially available iron chelators due to its oral availability and relatively low toxicity. Although the precise mechanisms by which deferasirox exerts its anti-cancer effects are still being investigated, we hypothesized that deferasirox inhibits cell cycle progression based on previous studies [11, 17]. We found that deferasirox induced G1 arrest by upregulating p21 and p27 and downregulating cyclin D1 and CDK4. These findings support our hypothesis that deferasirox exerts its anti-neoplastic effects by regulating cell cycle progression.

Iron chelators can induce the expression of NDRG1, a known metastatic suppressor, in a variety of human cancers [18–20]. The mechanism by which NDRG1 suppresses metastasis is presently unclear, although NDRG1 has been shown to inhibit cell migration and invasion by modulating the expression of a number of adhesion molecules [21]. NDRG1 expression has been shown to be significantly lower in cancer tissue compared with adjacent normal tissue; moreover, NDRG1 expression has been shown to be inversely correlated with the metastasis of some cancers, such as prostate and colorectal cancer [22, 23]. However, discrepant results have been obtained regarding a possible association of NDRG1 with tumor progression. Interestingly, NDRG has a demonstrated role in cell cycle control. Specifically, NDRG1 expression is upregulated via p53-mediated induction, and NDRG1 can also induce G1/S arrest by upregulating p21 [24]. We found that deferasirox upregulates the expression levels of NDRG1, p53, and p21. Although we did not assay cell migration or investigate metastasis in vivo in the present study, our findings suggest that deferasirox may be able to inhibit tumor growth and metastasis. We hypothesize that the mechanism by which deferasirox exerts its anti-tumor effects may involve NDRG1.

Deferasirox has been shown to enhance the cytotoxic effect of cisplatin in esophageal cancer cell lines. In addition, cisplatin-resistant cells treated with a low concentration of deferasirox (5 μM) in combination with cisplatin showed a significant reduction in cellular viability compared with cells treated with deferasirox or cisplatin alone [10]. We found that the combination of deferasirox (5 μM) and cisplatin (5 μM) induced a significant decrease in cellular viability. Moreover, this combined treatment resulted in the upregulation of NDRG1, p21, and p53 and the downregulation of phospho-mTOR. Our results, therefore, suggest that deferasirox can potentially enhance the anti-cancer effect of cisplatin in gastric cancer cells. Moreover, the p53-NDRG1-p21 and mTOR pathways may be involved in the synergistic effect of deferasirox with cisplatin.

This study did have a number of limitations. Firstly, we restricted our study to gastric cancer cell lines and did not perform any in vivo experiments. In addition, the expression of cell cycle-related proteins was assessed only by Western blotting. More detailed information could have been obtained by immunoprecipitation and kinase assays. To determine the anti-tumor effect of deferasirox, additional experiments including in vivo study would be needed. Nevertheless, this is the first study to investigate the anti-tumor effects of deferasirox against gastric cancer.

## Conclusions

In conclusion, we found that deferasirox induced anti-tumor effects in gastric cancer cells via various pathways. Specifically, deferasirox upregulated NDRG1, inhibited cell cycle progression, downregulated mTOR and c-myc expression, and induced apoptosis. Moreover, deferasirox potentiated the anti-cancer effects of cisplatin. Although the efficacy of deferasirox must be confirmed in future studies, our results indicate that deferasirox is a promising anti-cancer therapeutic agent and may also be an effective chemotherapy sensitizer.
